# Towards embedding Caco-2 model of gut interface in a microfluidic device to enable multi-organ models for systems biology

**DOI:** 10.1186/s12918-019-0686-y

**Published:** 2019-03-05

**Authors:** Dmitry Sakharov, Diana Maltseva, Evgeny Knyazev, Sergey Nikulin, Andrey Poloznikov, Sergey Shilin, Ancha Baranova, Irina Tsypina, Alexander Tonevitsky

**Affiliations:** 1 SRC BioClinicum, Moscow, Russia; 2 School of Systems Biology, George Mason University, Fairfax VA, USA; 3 Research Center of Medical Genetics, Moscow, Russia; 4 Department of Cell Biology, Higher School of Economics, Moscow, Russia; 5 Art photonics GmbH, Berlin, Germany

**Keywords:** Caco-2 cell lines, 2D layer, Microfluidic chip, On-the-chip models

## Abstract

**Background:**

A cancer cell line originating from human epithelial colorectal adenocarcinoma (Caco-2 cells) serves as a high capacity model for a preclinical screening of drugs. Recent need for incorporating barrier tissue into multi-organ chips calls for inclusion of Caco-2 cells into microperfused environment.

**Results:**

This article describes a series of systems biology insights obtained from comparing Caco-2 models cells grown as conventional 2D layer and in a microfluidic chip. When basic electrical parameters of Caco-2 monolayers were evaluated using impedance spectrometry and MTT assays, no differences were noted. On the other hand, the microarray profiling of mRNAs and miRNAs revealed that grows on a microfluidic chip leads to the change in the production of specific miRNA, which regulate a set of genes for cell adhesion molecules (CAMs), and provide for more complete differentiation of Caco-2 monolayer. Moreover, the sets of miRNAs secreted at the apical surface of Caco-2 monolayers grown in conventional 2D culture and in microfluidic device differ.

**Conclusions:**

When integrated into a multi-tissue platform, Caco-2 cells may aid in generating insights into complex pathophysiological processes, not possible to dissect in conventional cultures.

## Introduction

Current methodology for evaluating novel drug candidates in preclinical models is far from being perfect due to the difference in the metabolism of laboratory animals and humans, the limitations of single component in vitro systems and the human body, and the restrictions of in silico modeling [1–3].

The main goal of microfluidic organotypic chips is the emulation of human physiology on a miniature scale. As an in vitro approach with a great predictive power for human drug response it has a potential in the reduction of costly failures in the evaluation of drug efficacy and safety [4]. Microfluidic devices are designed to culture the cells under continuous perfusion and physiological shear stress in order to recapitulate the functions of the cells in culture, taking into account the specific tissue- and organ-level circulatory conditions [5]. Recently, multi-organ platforms, also known as “human-on-a-chip”, started to emerge as viable alternative for the laboratories involved in modeling of human diseases and drug development including target-based screening, and phenotypic screening [6]. These platforms open the avenue toward true systems biology driven modeling of the both normal human physiology [5, 7] and various pathological processes [8].

These are some necessary requirements for “human-on-a-chip” systems. First of all, these systems should be superior to the organotypical single organ equivalents, secondly, they should reflect systems biology of a human organism at a smaller scale by retaining functional connectivity of cultured organ and tissue representatives. A few multi-compartment microfluidic systems, which provide the simultaneous co-culture of different tissues, were described previously [9–12]. A majority of such systems expose the cells to laminar flow within microchannels [10, 13, 14]; this fluid flow may be adjusted to control local tissue-to-fluid ratio in the channels. Systems of this kind are burdened with some inherent disadvantages, such as a relatively small cell count and a significant dilution, resulting in a reduced molecular crosstalk between the tissues. As an example, Guzzardi et al. [15] in his work has reported the tissue crosstalk in a system hosting 2.4–6 10^5^ liver cells and 8–6 10^4^ endothelial cells in separate compartments and a total circulating media volume of 30 ml. Due to the use of external pumps and external media reservoirs embed in described system, tissue volumes represent only a tiny part of the overall circulating media.

We have met the tissue-to-media volume challenge by integrating a peristaltic micropump and media reservoirs into a multi-organ-chip (MOC) of a standard microscope slide. Therefore, a minimized fluid-to-tissue ratio within the whole system was achieved, while ensuring control of a media velocity. There have been several designs produced, of which the two-tissue MOC design has proven the capability of the MOC platform to simultaneously maintain a human liver equivalent and a human skin biopsy. The selection of these two tissues has been guided by utility considerations as the liver is considered to be the prime target for toxicity of pharmaceutical drug candidates, while human skin is the target tissue for cosmetics. As a critical need of incorporating barrier tissue into MOC devices has been increasingly recognized [16], the next generation of MOC design is being built to include the colon cancer Caco-2 cell line, widely used as a model for the intestinal epithelium. While been grown on permeable membranes it forms a monolayer of polarized epithelial cells, similar in phenotype to cells of the small intestine, which gives an opportunity to study their barrier, transport and secretory functions. The imitation of natural conditions in a microfluidic device with a constant circulation of the nutrient medium allowed us to obtain a more sophisticated model of the intestinal barrier, with a hope to recreate gut microenvironment and microbial flora [17, 18], investigate of the intestine barrier and transport function [19], immune response [20] and susceptibility to drugs [21].

This article describes a series of systems biology insights extracted from Caco-2 models cells grown in a 2D layer and in a microfluidic chip. The aim was to chart a way for integration of Caco-2 component into multi-organ platforms, and in validating existing intestine on-the-chip models.

## Results

### Simple tests do not differentiate a monolayer from microcirculation model

**Fig. 1:**
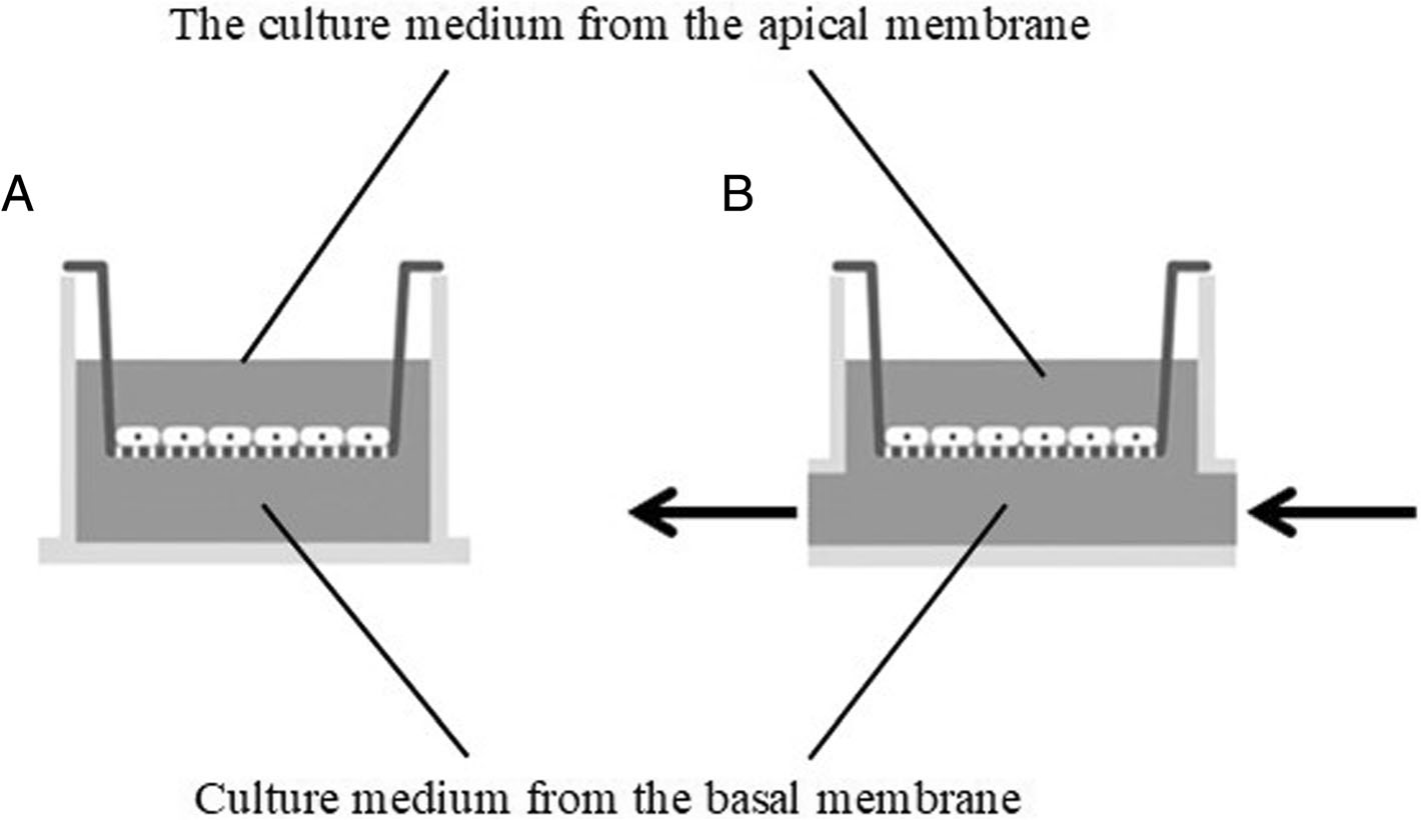
Scheme of obtaining culture media for the study of miRNA. **a** conventional conditions. **b** microcirculation conditions, arrows indicate the direction of current of the culture medium through microfluidic channels

To assess the state of the Caco-2 cells’ monolayer during the differentiation process, the basic electrical parameters of the monolayer were evaluated using impedance spectrometry (Fig. 1). The graphs in Fig. 1 show that by the 7th day of differentiation, the Transepithelial electrical resistance (TEER) values of the cells grown in a conventional monolayer condition and in a microfluidic device did not differ significantly (4590 ± 307 and 4815 ± 778 Ohms, respectively). These TEER values indicate the integrity of a monolayer corresponding to a good barrier function, differentiated polarized cells with formed tight junctions. Between the differentiation days 3 and 7, the values of the electrical capacitance C increased by approximately 2.8–2.9 times, indicating an increase in the area of cell membranes due to the formation of microvilli on the apical surface of Caco-2 cells during differentiation [22]. The absence of significant differences between the conventional and the microcirculation conditions demonstrates similar viability of the monolayer in both cultivation modes. The MTT test confirms lack of significant differences in cell viability.

**Fig. 2:**
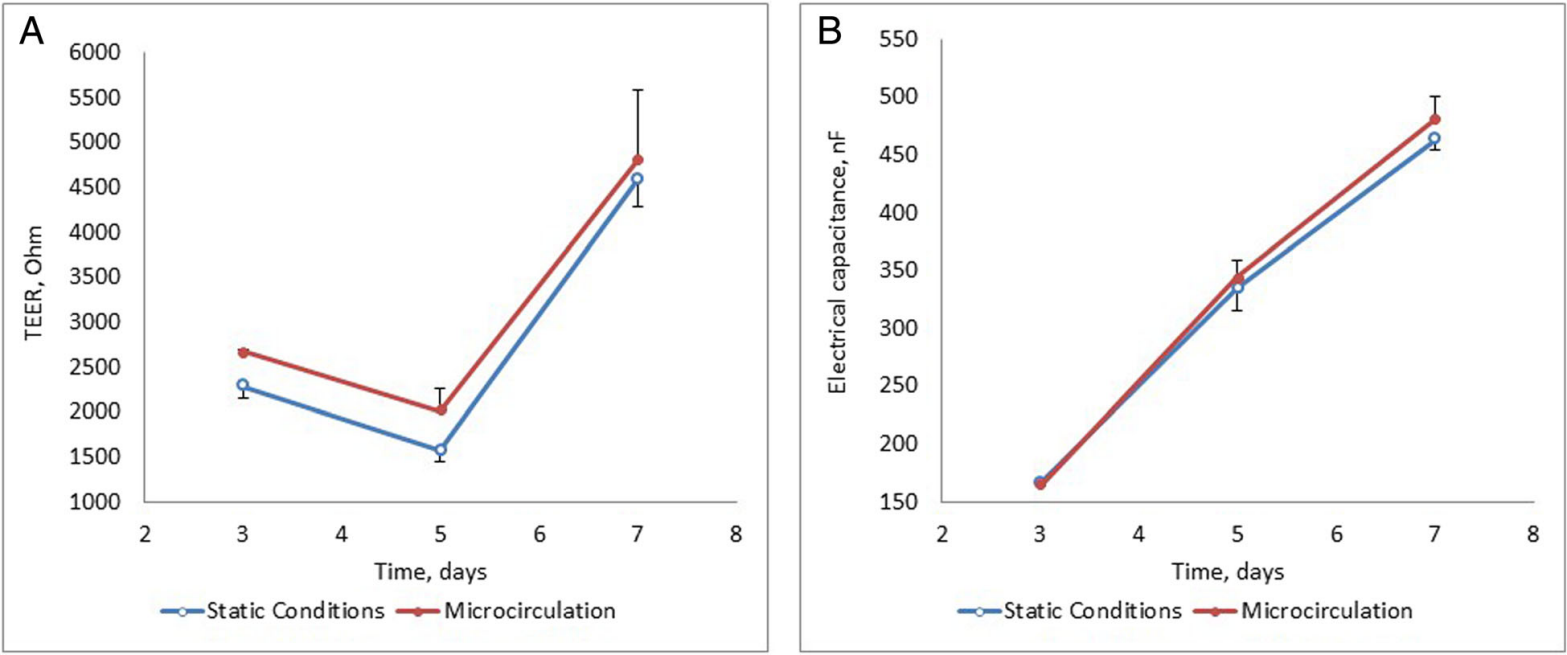
Changes in the electrical parameters of a monolayer of cells assessed using the Impedance Spectrometry System (SRC BioClinicum). **a** the dynamics for changes in transepithelial electrical resistance TEER, **b** the dynamics of changes in electrical capacitance C

In a subsequent experiment, the pre-formed confluent monolayers composed of 98 differentiated Caco-2 cells were incubated either under conventional conditions or in a 99 three-well microfluidic chip [21]. One of the wells contained the Transwell® unit with cells, while nutrients and metabolites were constantly equilibrated within the circuit by microcirculation. No significant changes in cell morphology were observed; monolayers under conventional culture conditions (Fig. 2a) and under microfluidic conditions in a chip (Fig. 2b) remained intact until the end of the incubation.

Nevertheless, more detailed investigations showed that the impression that everything is fine with the monolayer grown in conventional conditions is incorrect.

### Expression profiles of CACO-2 cells grown as monolayer in conventional conditions differ from those grown in conditions of physiological microcirculation

Metabolic properties of cells cultured under different flow conditions and the inherent variation in drug response heavily contribute to commonly reported fluctuations in predictive power of preclinical model system. Additionally, depending on cultivation conditions, the selection for the growth of particular subpopulations of cells may result in a variety of cellular model systems with properties that may differ from the original cell line [23]. Because of all three factors, results obtained under similar experimental conditions in different laboratories may not be directly comparable. Among commonly observed variations in genome-wide transcription landscapes, the most pertinent differences are expected to disturb so-called druggable genome, defined as the genes or gene products known or predicted to interact with drugs, carefully curated and placed in publicly available Drug-Gene Interaction Database (DGIdb) [24, 25]. In in silico experiment described below, we compared “druggable transcriptomes” of Caco-2 monolayers grown in microcirculation devices or in conventional conditions [26]. Expression levels of a total of 23 druggable genes were consistently low in Caco-2 cells grown either as in microfluidic chip and in tumors (*p*-value < 0.0002). It is noteworthy that the mentioned genes encode the proteins responding to 57 different small molecule drugs, with the most abundant activity classes being anticholesteremic and antineoplastic ones.

This study shows that the druggable gene expression profiles of Caco-2 cells grown in microfluidic chip resemble ones observed in Caco-2 formed tumors, while substantially differing from that in conventional monolayer conditions. This observation indicates that conventional Caco-2 studies may overestimate the power of particular drug candidate to modulate a molecular target, thus, emphasizing the necessity of expanding the use of microfluidic devices chip in a drug discovery process.

### A comparison of laminin expression indicates subtle differences in degree of cell differentiation and polarization within CACO-2 cells grown in conventional conditions and conditions of microcirculation

The signals received by the cell from the components of the basal membrane play an important role in the formation and maintenance of the epithelial tissues’ architecture [27]. Laminins consist of three types of subunits, α, β, and γ, with 5 types of α-chains, 4 of β-chains, and 3 of γ-chains, which together form 18 different laminins. However, each subunit in the composition of the trimer may have a different signal function. When the mRNA level encoding various chains of laminins were analyzed in Caco-2 cells differentiating under microcirculation and in conventional culture, significantly lower expression of the *LAMA1* gene, encoding laminin-α1, was detected in the former (*p* = 0.022) [28].

Laminin-α1 is highly expressed in embryonic tissues. As mature epithelial tissues are formed and epithelial cells differentiate, the levels of the production of this chain drop [27]. Lower expression of *LAMA1* observed in cells completing their differentiation under microcirculation indicates that microfluidic devices provide more favorable conditions for the polarization of the epithelium.

### Microcirculation in chip modulates EXPRESSION of cell adhesion genes involved in progression of colon carcinoma

Caco-2 is a cancer cell line originating from human epithelial colorectal adenocarcinoma [29]. This cell line is of a unique use, as most of the drugs are resorbed in the small intestine. Along with other models, this cell line has found its use in a various studies of colon carcinoma, including those aimed at understanding of its basic biology and at the search for novel molecular targets [30–32].

The progression of colon cancer is usually forced by epithelial-mesenchymal transformation of tumor cells, where the role of cell adhesion molecules (CAMs) is critical [33, 34]. The expression profiles of CAM-encoding genes in Caco-2 colon cancer cells grown in conventional conditions and in a microfluidic chip differ (see Table 1). In particular, microfluidic perfusion stimulates expression of genes coding glycoproteins CEACAM5 and CEACAM6, prototype biomarkers for colon carcinoma [35–37]. The list of other genes upregulated under dynamic microcirculation conditions included one for glycoprotein CD44, a surface marker for colon cancer stem cells [38–40]. The adhesiveness of cancer cells is historically known to be of special interest. This property is of special interest in tissues which are subject to shear stress like colon in contrast to non-shear stress epithelia [41]. The list also included one for cadherin 7, involved in calcium-dependent cell-cell adhesion [35], one for L1CAM, overexpression of which in colorectal tumor was previously associated with shorter survival [42], one for proteoglycan versican and one for CYR61, cysteine-rich angiogenic inducer 61, known biomarkers of colorectal cancer [43, 44]. For the latter two genes, expression levels were found higher in primary tumor cells as compared to advanced cancers [35, 43], possibly indicating that microfluidic-chamber cultured Caco-2 cells emulate relatively early stages of tumor development. The list of genes down-regulated in microfluidic chip as compared to conventional culture, included COL12A1, which encodes alpha chain of type XII collagen [45], and two shear stress vulnerability genes *GJA1* and *ITGA5* [46, 47].

**Table 1:** MicroRNAs, the level of which significantly increased in the culture medium over the Caco-2 cells when cultivated under conventional conditions for 24 h

miRNA	Log_2_-level for zero	Log_2_- level for the medium under statistic conditions	Expansion rate	*p*-value	Log_2_-level in cells under statistic conditions
hsa-miR-200c-3p	0,4	4,8	21,6	< 0,001	10,2
hsa-miR-378a-3p	0,9	4,6	12,5	0,001	7,9
hsa-miR-194-5p	0,9	4,2	9,9	0,010	10,3
hsa-miR-192-5p	0,8	3,4	6,2	0,001	9,5
hsa-miR-103a-3p	1,1	2,9	3,7	0,047	8,9
hsa-miR-210-3p	0,7	2,5	3,6	0,048	6,9
hsa-miR-24-3p	1,1	2,9	3,4	0,020	7,4
hsa-miR-106a-5p	1,4	2,9	3,0	0,037	9,4
hsa-miR-320a	3,4	4,9	2,7	0,049	7,9
hsa-miR-222-3p	0,8	2,2	2,6	0,005	7,2
hsa-miR-17-5p	1,8	3,1	2,5	0,049	9,6
hsa-miR-221-3p	1,3	2,6	2,3	0,020	7,0

### PERFUSION-RELATED SWITCH IN CELL ADHESION ENCODING GENES IS MEDIATED BY miRNAs

**Fig. 3:**
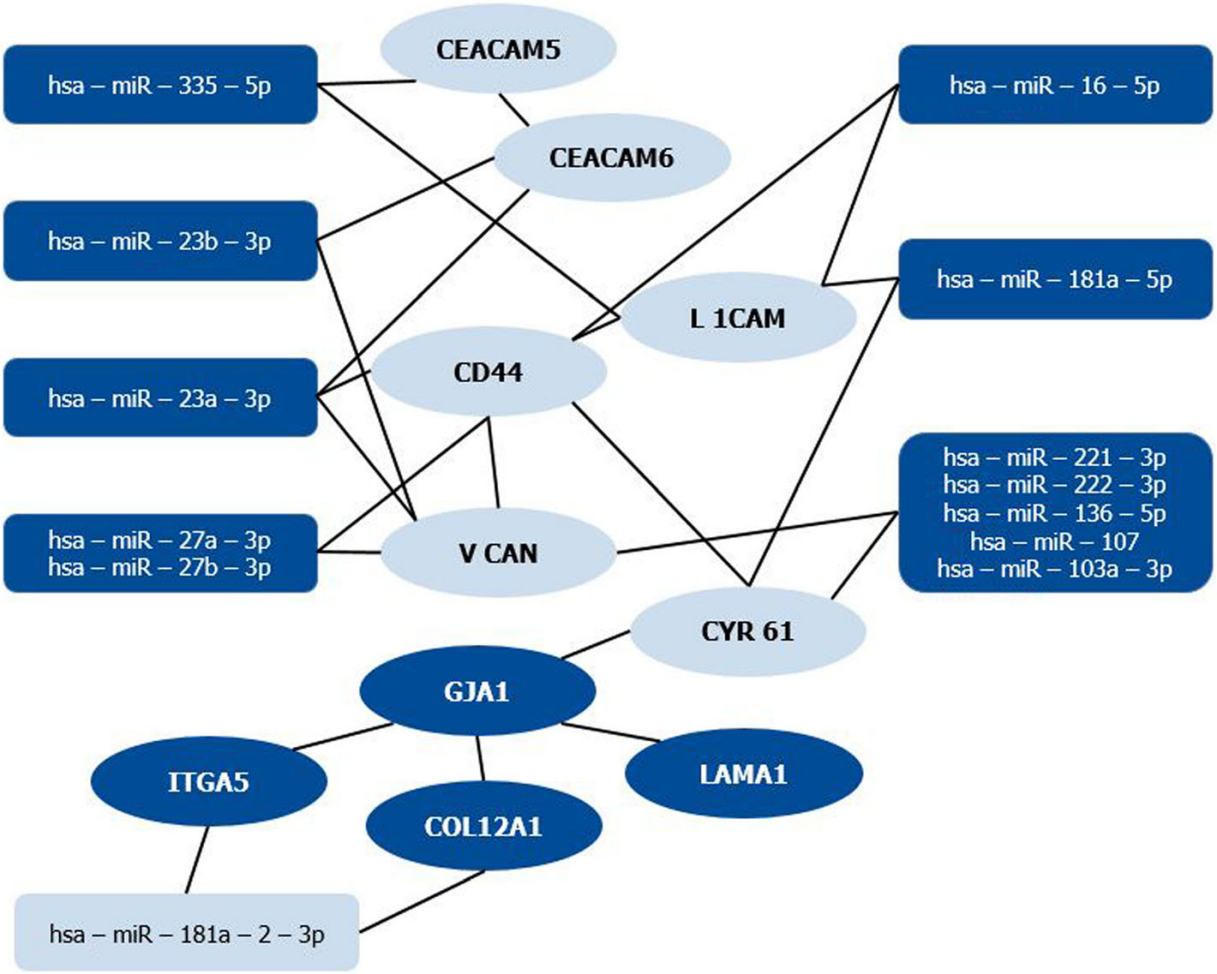
Regulatory network of CAM mRNAs-miRNAs. The up-regulated genes and miRNAs are colored with light blue, down-regulated genes and miRNAs are colored with dark blue

When the miRNAs with the largest changes in their expression levels in response to microcirculation were investigated for their possible intracellular targets, the network of cell adhesion molecules (CAMs) was identified as visibly affected. The core part of the network is formed by CD44 and GJA1 acting as two hubs with a notable interconnectivity (Fig. 3). Further analysis highlighted 12 miRNAs as contributing to this network through regulation of more than one cell adhesion gene (Table 1, Fig. 3). 12 miRNAs regulate the expression of CAM genes, namely hsa-miR-181a-2-3p (genes *COL12A1* and *ITGA5*), hsa-miR-16-5p (genes *CD44* and *L1CAM*), hsa-miR-335-5p (genes *CEACAM5* and *L1CAM*), hsa-miR-23b-3p (genes *CEACAM6* and *VCAN*), hsa-miR-181a-5p (genes *CYR61* and *L1CAM*). Two miRNAs (hsa-miR-27a-3p and hsa-miR-27b-3p) target genes *CD44* and *VCAN* at the same time. *CYR61* and *VCAN* are regulated by five miRNAs (has-miR-221-3p, hsa-miR-222-3p, hsa-miR-136-5p, hsa-miR-107 and hsa-miR-103a-3p). All these miRNAs were down-regulated in microcirculatory conditions except for hsa-miR-181a-2-3p which suppresses expression of alpha chain of type XII collagen and integrin alpha 5 (Fig. 3). A majority of CAM-encoding genes were targeted by several miRNAs, with some interacting gene pairs being targeted by the same set miRNAs within the network (Fig. 3). It is of note that hsa-miR-181a-2 [48], hsa-miR-375 [49], hsa-miR-206 [50] and hsa-miR-129-5p [51] and some other miRNAs have already been shown as associated with cancer phenotypes. Presented network emphasizes an importance of cell adhesion in a functioning of a perfused intestine model (Fig. 3).

### Pools of MIRNAS secreted at apical side of the CACO-2 membranes grown in conventional culture and under microcirculation differ

For conventional and microperfused Caco-2 cultures, intracellular miRNA pools and the culture medium samples collected from apical and basal compartments were profiled and compared (Fig. 2). The media collected form apical side of Caco-2 cells grown in conventional conditions showed a total of 12 secreted microRNAs (Table 1), while microperfused cells overexpressed 5 miRNA species (Table 2), all of which were also present in the media conditioned by cells grown in conventional conditions. All 12 secreted by conventionally grown Caco-2 cells displayed a high signal in intracellular pools (Tables 1, 2), among the Top 30 miRNAs with the highest signal. This may indicate that in conventionally grown Caco-2 cells, miRNAs may enter the culture medium both through selective secretion and through non-specific release, proportional to its concentration in the cell itself, possibly highlighting relative loss of membrane integrity in non-perfused Caco-2 monolayer.

**Table 2:** MicroRNAs, the level of which significantly increased in the culture medium over the Caco-2 cells when cultured under microcirculation conditions for 24 h

miRNA	Log_2_-level for zero	Log_2_- level for the medium under microcirculation	Expansion rate	p-value	Log_2_-level in cells under microcirculation
hsa-miR-200c-3p	0,4	3,7	9,9	0,003	9,1
hsa-miR-192-5p	0,8	3,5	6,4	0,006	8,7
hsa-miR-210-3p	0,7	2,4	3,4	0,005	5,8
hsa-miR-194-5p	0,9	2,3	2,6	0,019	9,4
hsa-miR-378a-3p	0,9	2,0	2,1	0,031	7,1

One of the miRNAs that remained at a stable level under microcirculation, hsa-miR-320a, was previously reported as elevated in the blood of mice with experimental colitis and of patients with Crohn’s disease. The gut epithelium cell line can secrete hsa-miR-320a as a protective reaction to the effects of pro-inflammatory agents, tumor necrosis factor α (TNF-α) and interleukin 1β (IL-1β) [52]. Two other miRNAs that followed the same trend, miR-24-3p and miR-221-3p microRNA levels, are increased in the intestinal cells of an animal model of irritable bowel syndrome, which is associated with the development of inflammation of the intestinal mucosa of low severity [53]. It is possible that an increased secretion of these miRNAs by Caco-2 cells under conventional conditions may indirectly indicate the activation of the inflammatory pathways of the cell in response to stress in the absence of medium circulation.

## Discussion

Microcirculation leads to a decrease in the expression of laminin α1 (LAMA1), and a coordinated change in expression of many other CAM-encoding genes. The network analysis highlights that observed expression changes are coordinated by miRNAs. Moreover, observed differences of the sets of miRNAs secreted at the apical surface of Caco-2 monolayers grown in conventional 2D culture and in microfluidic device with intracellular population of these molecules point at possible contribution of the direct leakage of intracellular miRNAs to secretome of conventionally cultured monolayers. These observations indicate that the differentiation and the polarization of the cells in the presence of medium circulation are more complete than that observed in conventional culture. This study stresses the shortcomings of non-perfused cellular models, and highlights the necessity of proper microcirculation to be built into the MOC devices.

## Conclusion

Cell lines have proved themselves not only as high capacity screening models or biopharmaceutical assessments (like ADMET, potency properties), but also as a framework for the evaluation of transport mechanisms useful both in pre-clinical and clinical phases of the drug discovery. Models based on Caco-2 cells form cost effective, quick and easy in vitro methods, which form high throughout, reliable and reproducible data. Unfortunately, when grown in a monolayer Caco-2 cells resemble human guts to a degree at best, as their capacity to participate in molecular crosstalk among tissues is limited [54–58]. On the other hand, when integrated into a multi-tissue platform, Caco-2 cells may assist in generating insights useful for the understanding of complex pathophysiological processes, e.g. inflammatory organ crosstalk.

## Materials and methods

### Cell culture

The Caco-2 cells were obtained from the Russian Vertebrate Cell Culture Collection (Institute of Cytology, Russian Academy of Sciences, Russia). Undifferentiated cells were seeded on individual polyether membrane inserts with 0.143 cm2 surface area and 1.0 μm pore diameter, cut out from HTS Transwell®-96 well permeable support (Corning Inc., USA), with cell density approximately 60,000 cells/well. Cell counts were performed with automated cell counter Countess (Invitrogen, USA). The cells were incubated under conditions for differentiation for 7 days in MEM with 10% FBS, 0.1 mM non-essential aminoacids, 0.1% penicillin-streptomycin (all reagents from Gibco, USA) in 5% CO2, 37 °C, changed three times. Transwell® inserts with Caco2 monolayers were either kept on the microplate or put into microfluidic chip. After 24-h incubation the cells were lysed in 700 μl of Qiazollysis reagent (Qiagen, Germany) and subjected to microarray expression analysis. The experiments were performed in triplicates.

### Microfluidic chip

The microfluidic chip consisted of 3 layers: 10 mm polycarbonate plate having the wells closed by lids and ports for pneumatic and fluidic connections, 2 mm polydimethylsiloxane (PDMS) layer containing the microfluidic circuit and wells, and standard 1-mm microscopic glass slide as a basis for the chip. The total volume of the chip was 1300 μl. The well #3 contained a single element of HTS Transwell®-96 well permeable support (Corning Inc., USA) with a pre-formed Caco2 monolayer. The chip had an integrated peristaltic pump comprising pneumatic-actuating valves in the circuit and controlled by proprietary Hemule control unit with the flow rate range from 0 to 40 μl/min.

To run circulation mode in the circuit, the valves 1 and 3 were closed, and 6-step algorithm included the following actions on the inlet valve 2, the expansion chamber 4 and the outlet valve 5: pressure, pressure, pressure; vacuum, pressure, pressure; vacuum, vacuum, pressure; pressure, vacuum, pressure; pressure, vacuum, vacuum; pressure, pressure, vacuum. The parameters of a microcirculation pump were set for ±20 kPa and 2 Hz resulting in the medium flow rate of 4.13 μl/min and constant concentration of nutrients in the wells due to advection.

### Microarray analysis

RNA isolation and quality controls from incubated cells as well as microarray expression analysis were performed. The procedures for cDNA synthesis and labelling were carried out according to the Ambion WT Expression Kit (Life Technologies, Darmstadt, Germany) using 500 ng total RNA as starting material. These experiments were performed using Affymetrix Human Gene 1.0 ST Array (Affymetrix Inc., Santa Clara, CA, USA).

### Bioinformatic analysis

For the identification of genes microarray datasets GSM802198, GSM802199, GSM8022007 and GSM1031890, GSM1031891, GSM10318926 were jointly preprocessed in AffymetrixConsole software using RMA method and the resulting expression levels were analyzed using AffymetrixTranscriptome Analysis Console. All probesets with no associated gene symbol were excluded from the analysis. Probesets with mean expression level in each of the compared groups not exceeding 128 (i.e., 7 log-scaled) were also excluded. The differential expression thresholds were set to 1.5x for fold change and to 0.05 for *p*-value. A gene was classified as differentially expressed if there was at least one probeset associated with this gene that demonstrated higher expression level in one group compared to the other group (fold change above 1.5x and *p*-value lower than 0.05), and there were no probesets associated with this gene that demonstrated the opposite difference (expression level in the other group higher in comparison with the first group with fold change above 1.5x and p-value less than 0.05).

The same procedure and the same thresholds were applied to our microarray expression data used for identification of differentially expressed genes between static cell culture and microfluidic chip.

The list of druggable genes, encoded proteins and drugs was taken from the Drug-Gene Interaction Database (DGIdb). The analysis of differentially expressed druggable genes was focused on the genes that had lower expression in case of cultivation within a microfluidic chip with circulation when compared to conventional cell culture.

The Binomial test was used to obtain the upper bound for p-value associated with the fact that a gene set is randomly distributed between groups of genes which are up- or down-regulated compared to conventionally cultured cells.

Heat maps were constructed using R package “pheatmap”, and show relative log-scaled expression values for each gene (i.e., differences between log-scaled expression values and mean log-scaled expression observed for this gene in all depicted samples).

### Evaluation of in vitro intestinal barrier function

Evaluations of the in vitro barrier function were performed in three ways: using a model substrate, using a TEER measurement and using the measurement of impedance spectra.

The fluorescent luciferic yellow dye was used as a model substrate. This dye is commonly used to assess the integrity of in vitro models of the intestine, as it is transported only through passive diffusion [28] [59]. After 3 weeks of cultivation each membrane was washed 2 times with Hanks salt solution (100 μl for the apical part, 235 μl for the basal part). Then, 100 μl of Lucifer Yellow solution with a concentration of 60 μM was added to the apical part (Hanks salt solution was used as the solvent, the final concentration of DMSO was brought to 1% by volume), and to the basal solution 235 μl of Hanks salt solution from 1% DMSO by volume was added. Then the plate was incubated in a cell incubator (5% CO2, 37 C) for 1 h. The final concentration of luciferous yellow in the apical and basal portions was determined using a SpectraMax i3 tablet multi-detector (Molecular Devices). The method of calculating the permeability coefficient according to the data on the fluorescence intensity of a substrate that has passed through the cell layer has been described previously [60].

The TEER measurements were carried out at room temperature using an STX100C96 electrode (World Precision Instruments) and an EVOM volt-ohm meter instrument (World Precision Instruments) [61].

Impedance spectra were measured in the frequency range from 20 Hz to 20 kHz using an impedance spectrometry system (SRC BioClinicum) and STX100C96 (World Precision Instruments) electrode at room temperature.
